# “Not by Our Feeling, But by Other's Seeing”: Sentiment Analysis Technique in Cardiology—An Exploratory Review

**DOI:** 10.3389/fpubh.2022.880207

**Published:** 2022-04-11

**Authors:** Adrian Brezulianu, Alexandru Burlacu, Iolanda Valentina Popa, Muhammad Arif, Oana Geman

**Affiliations:** ^1^Faculty of Electronics, Telecommunications and Information Technology, “Gheorghe Asachi” Tehnical University, Iaşi, Romania; ^2^GreenSoft Ltd., Iaşi, Romania; ^3^Faculty of Medicine, “Grigore T. Popa” University of Medicine and Pharmacy, Iaşi, Romania; ^4^Department of Interventional Cardiology - Cardiovascular Diseases Institute, Iaşi, Romania; ^5^Department of Computer Science and Information Technology, University of Lahore, Lahore, Pakistan; ^6^Neuroaesthetics Laboratory, “Ştefan cel Mare” University, Suceava, Romania

**Keywords:** sentiment analysis, cardiovascular, artificial intelligence, machine learning, social media

## Abstract

Sentiment Analysis (SA) is a novel branch of Natural Language Processing (NLP) that measures emotions or attitudes behind a written text. First applications of SA in healthcare were the detection of disease-related emotional polarities in social media. Now it is possible to extract more complex attitudes (rank attitudes from 1 to 5, assign appraisal values, apply multiple text classifiers) or feelings through NLP techniques, with clear benefits in cardiology; as emotions were proved to be veritable risk factors for the development of cardiovascular diseases (CVD). Our narrative review aimed to summarize the current directions of SA in cardiology and raise the awareness of cardiologists about the potentiality of this novel domain. This paper introduces the readers to basic concepts surrounding medical SA and the need for SA in cardiovascular healthcare. Our synthesis of the current literature proved SA's clinical potential in CVD. However, many other clinical utilities, such as the assessment of emotional consequences of illness, patient-physician relationship, physician intuitions in CVD are not yet explored. These issues constitute future research directions, along with proposing detailed regulations, popularizing health social media among elders, developing insightful definitions of emotional polarity, and investing research into the development of powerful SA algorithms.

## Introduction

Sentiment Analysis (SA) or “opinion mining” is a novel branch of Natural Language Processing (NLP) that measures emotions or attitudes behind a written text. At the most basic level, SA tools classify pieces of text as having positive, negative, or neutral emotions, although current technologies support much more complex analysis of emotions in the written text (1).

Several artificial intelligence (AI)/machine learning (ML) technologies and other types of computational techniques have been proposed and proved their benefits in bettering diagnostic accuracy and treatment efficacy (2). The existence of intuition and the documentation of its importance in patient management urges the enrichment of medical AI/ML and other computational methods with the ability to detect and assess emotions to attain higher performance in solving health problems. Using SA to examine doctors' written notes on intensive-care-unit patients, the paper showed that doctors' intuitions (“gut feelings”) were an essential factor in determining the disease management for each patient (3).

To further explore the effectiveness of SA in medical context, a review investigating the SA methods used for examining emotions in healthcare tweets has been published (4). However, no review has focused, so far, on evaluating the utility of SA in cardiology. Cardiovascular diseases (CVD) arouse a particular interest as they are the deadliest diseases in the world (5). Recent studies suggest that SA could be extremely useful in cardiology, especially in the context of extensive use of telemedicine due to the COVID-19 pandemic (6).

The increasing use of social platforms may be the foundation for developing SA-based models applied to various fields in cardiology. A study on drug safety showed that adding SA features improves the performance of state-of-the-art methods to identify adverse drug reactions (ADR). These models used a corpus of posts from Twitter and other online forums. SA features significantly increased the F-measure of adverse reaction detection (for 81 drugs, including cardiovascular medication) from 72.14 to 73.22% in the Twitter corpus of posts. The improvement of ADR detection by SA became possible due to the rapidly growing popularity of social media and health forums (7, 8).

Our narrative review aims to: (1) summarize the current directions of SA in cardiology and the results achieved so far in a systematic manner, (2) raise the awareness of cardiologists about the potentiality of this novel and promising domain that will soon become a practical reality, and (3) open new perspectives regarding the dialogue between AI specialists and cardiologists. Given the small number of studies so far, a systematic methodology is not suitable, therefore the purpose of this review is purely narrative.

## Materials and Methods

We searched PubMed/Medline and Google Scholar for studies in English addressing the topic of SA in cardiology, from inception to february 2022. The following search string was used: (“Sentiment analysis” OR “Emotions recognition” OR “Sentiment recognition”) AND (“Heart failure” OR “Cardiac insufficiency” OR “Coronary Artery Disease” OR “CAD” OR “Coronary syndrome” OR “Coronary” OR “Stable angina” OR “Angina pectoris” OR “Ischemic heart disease” OR “IHD” OR “Ischemic” OR “Ischemia” OR “Myocardial infarction” OR “Infarction” OR “Atrial fibrillation” OR “AF” OR “Stroke” OR “Arrhythmia” OR “Heart rate” OR “Pulse” OR “Sudden death” OR “Sudden cardiac death” OR “Cardiovascular prevention”).

We reviewed an initial number of 550 studies, and after excluding the duplicates, 498 studies remained. After excluding the studies irrelevant to our objectives, we selected 11 papers that address SA methods focusing on cardiovascular diseases. Papers were included regardless of whether they constituted original research, reviews, opinions, reports. Any type of study was considered eligible for inclusion. Three researchers realized the agreement between the studies selected. All included studies are illustrated in Table 1.

**Table 1:** Characteristics of the included studies reporting SA solutions for cardiovascular diseases research.

**Authors**	**Objectives**	**Data sources**	**SA methods**	**Results**
**1. Detecting emotional risk factors for CVD**				
Eichstaedt et al., (9)	Analyze social-media language to identify community-level psychological correlates of age-adjusted mortality from AHD	Data from 1,347 US counties for which AHD mortality rates, health variables, and 50,000 tweeted words were available	Cross-sectional regression model based on Twitter language	Negativity emerged as significant risk factor (partial rs = 0.06, 95% confidence interval, or CI = [0.00, 0.11], to 0.12, 95% CI = [0.07, 0.17]) for CAD mortality
Hemalatha et al., (10)	Identify relevant MI risk factors using Twitter data	Twitter users with a MI history	LR for positive/negative emotion classification, with words weighted using TF.IDF	Not available
Medina Sada et al., (11)	Identify the relation between the sentiment of tweets and CVD	Tweets in the counties along Interstate 20 in Texas	Naïve Bayes, Multinomial Naïve Bayes, Bernoulli Naïve Bayes, Support Vector, and Linear Support Vector	High positive-to-negative ratio and positive-to-population ratio tend to associate with counties with low CVD rate
**2. Detecting positive/negative attitudes of CV patients toward their disease**				
Verma et al., (12)	Assess public health impact of CVD and patients' adherence and attitudes toward the disease	Tweets in english related to CVD	Not specified	The percentage of positive tweets are 45%, neutral tweets are 30 and 25% are negative tweets
Pimenta et al., (13)	Identify which fitness and nutrition apps that support behavior change (which could reduce CVD mortality) elicits a positive response from the users	User store reviews of a sample of fitness and nutrition apps	Text mining with Sketch Engine online app	StepsApp pedometer had the highest percentage of positive tags while VeryFitPro had the lowest
**3. Detection of cardiac arrhythmia**				
Behadada et al., (14)	Provides insights into arrhythmia detections from big data information sources	Expert knowledge, data and textual information from Pubmed articles and MIT-BIH database	Semi-automatically fuzzy partition rules and grammar-based text extraction SA	Accuracy of 93% and a high level of interpretability of 0.646 for the detection of cardiac arrhythmia
**4. Triage of CV patients**				
Lowres et al., (15)	Assessing the feasibility of using an ML program to triage incoming SMS text messaging replies as requiring health professional review or not	3,118 SMS text messaging replies received from 2 clinical trials	Naïve Bayes, OneVsRest, Random Forest Decision Trees, Gradient Boosted Trees, Multilayer Perceptron	The multilayer perceptron model achieved the highest accuracy (AUC 0.86)
**5. Feedbacks from patients and newspapers: reviews on drugs, therapeutic procedures, or medical devices**				
Pérez et al., (16)	Identify opinions on the drugs prescribed for chronic-degenerative diseases (including hypertension medication)	Blogs and specialized websites in the Spanish language	Hybrid approach (supervised machine learning and use of semantics through a tagged corpus)	The analysis of the sentiments of the opinions on the prescribed drugs is successful and reduces time and effort
Austin et al., (17)	Understand patients' attitudes toward LVAD therapy	Posts, comments, and titles from MyLVAD.com	Lexicon-based SA	Positive sentiment words are the most frequent. In comparison to other LVAD complications, “infection” is mentioned disproportionately more times.
Emerging Markets, (18)	Assess whether Biotricity (health tech company targeting mainly chronic CVDs) trends positively or not in the media	News media	InfoTrie Financial SA Solutions	Biotricity has been trending positively, achieving a news buzz score of 10 out of 10, with a market sentiment score of 4.0
**6. SA modules integrated in new technological concepts for monitoring CV patients**				
Sharma et al., (19)	Propose a smart conceptual framework for monitoring patients with CV or diabetes	Social media and other online resources (for the SA component)	Hybrid system merging SA techniques, data mining, ML, IoT, bio-sensors, chatbots, contextual entity search, granular computing	Not available

Cardiovascular disease (CVD); Atherosclerotic heart disease (AHD); United States (US); Coronary arteries diseases (CAD); Myocardial infarction (MI); Logistic regression (LR); Term Frequency * Inverse document frequency (TF.IDF); Left ventricular assist device (LVAD); Machine Learning (ML); Internet of Things (IoT).

The main directions of research regarding SA in cardiology identified from the retained studies are: the identification of emotional risk factors for CVD, the detection of positive/negative attitudes of CV patients toward their disease and its clinical implications, the detection of cardiac arrhythmia, the triage of CV patients, spotting feedback from patients and newspapers regarding drugs, therapeutic procedures, or medical devices and the integration of SA modules in new technological concepts for monitoring CV patients. Each of these topics is discussed below in an attempt to synthesize the current literature on SA in cardiovascular diseases, right after introducing the reader to basic concepts regarding medical SA and justifying how SA can contribute to increasing quality in cardiovascular healthcare.

## Medical Sentiment Analysis—Introductory Concepts

### What Is SA in Medicine?

Medical SA is the field of study that analyzes patients' and doctors' opinions, sentiments, attitudes, and emotions toward various clinical contexts (treatment side-effects, medical diagnosis concerns, emotional consequences of illness, emotional context during the onset or evolution of a specific disease, patient-physician relationship, physician attitudes in clinical notes) expressed in written text (20). While traditional AI deals with facts and logical, objective data analysis, sentiment research refers to opinions—correctly identifying subjective emotional communication.

Several medical entities associable with sentiments have been defined: health status (improved/worsened, good/bad), medical condition (improved/worsened), diagnosis (certain/uncertain/preliminary), medical procedure (positive/negative outcome), medication (helpful/useless/adverse events) (20).

### Sentiment Classification

Sentiment classification comprises two comprehensive categories: lexicon-based and ML/NLP-based classifications (21). The classifiers build upon sentiment lexicons (i.e., a collection of polar or opinion words, associated with their sentiment polarity, that is, positive or negative) are lexicon-based (or rule-based classifiers). Sentiment lexicons are produced manually or semiautomatically (22) and regularly stored as dictionaries. Conversely, ML/NLP-based classifiers are built using training datasets or annotated data collections.

### Types of SA

Some of the most popular types of SA are: fine-grained SA, emotion detection, aspect-based SA and multilingual SA (23). Fine-grained SA considers an expanded number of polarity categories (e.g., very positive / positive / neutral / negative / very negative). Emotion detection uses lexicons or ML/NLP systems to detect sentiments. Aspect-based SA highlights which particular aspects or features people are mentioning in a positive, neutral, or negative way (23). Multilingual SA techniques have been developed in order to analyses data in different languages (24).

**Figure 1:**
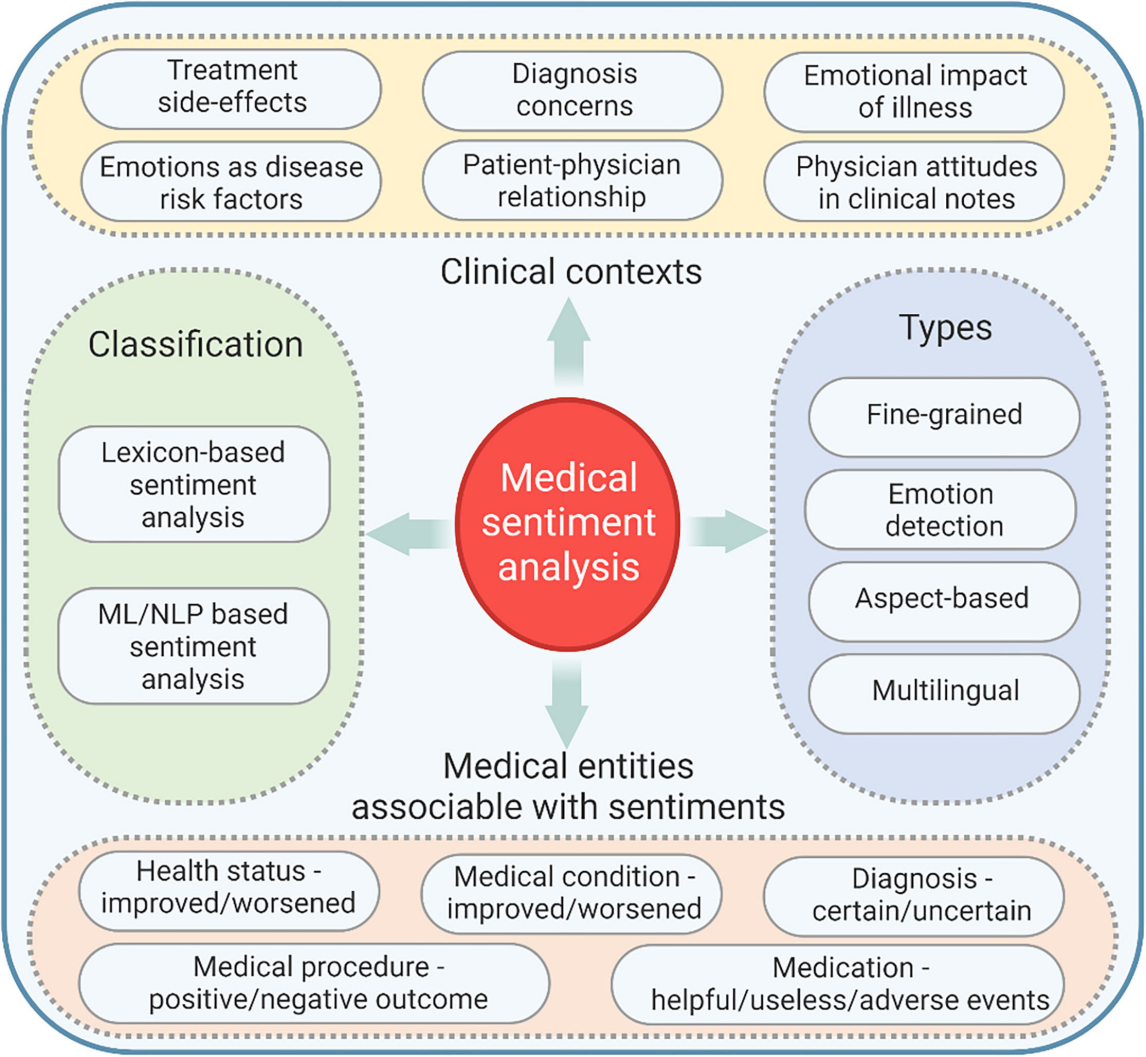
Introductory concepts in medical SA: applicable clinical contexts, medical entities associable with sentiments, classification, and types of SA.

The introductory concepts in medical SA are summarized in Figure 1.

## Justifying the Need to Integrate Modern Sentiment Analysis Solutions in Cardiology: How Sentiment Analysis Can Contribute to Increasing Quality in Cardiovascular Healthcare

CVD and emotional disorders seem to influence each other in a bidirectional manner (25). Coronary arteries diseases (CAD) and their impact in triggering emotional disorders is well documented. Moreover, emotional disturbances have the power to induce or worsen existing coronary artery diseases (25). Emotional disturbances were reported as potent cardiovascular (CV) risk factors (25).

There is a need to integrate the assessment of the emotional status in the cardiovascular risk prediction, a desideratum long considered unattainable due to a high degree of subjectivity regarding human sentiments and insufficient technical developments (25). The considerable technological and AI progress provides the opportunity to start developing strategies for building solutions capable of systematically assessing emotions. Since patients express their thoughts and feelings more openly in online than ever before (26), SA is becoming an essential tool to monitor and understand these sentiments and provide predictive models (27).

The increasing popularity of social platforms and discussion forums enabling the collection of unlimited amounts of written text and opinions, creates a favorable ground for testing novel SA methods. A descriptive study identified 4.9 million Tweets about CVD having common topics such as risk factors, awareness, and management of CVD (28). Using the vast amount of Twitter data on CVD, a study predicted county-level heart disease mortality based on the assessment of psychological language in Twitter posts (9). Given the SA superiority over standard predictive models (29), SA integration would lead to higher performance models and more complex CV predictions.

An obstacle to further improvement of AI/ML models is that 90% of the world's data is unstructured (30). While exam results are reported in a structured way, observations, intuitions, opinions, and experiences are communicated in an unstructured manner throughout clinical records, notes or online. Unstructured data is extremely time-consuming to analyses and it is unusable by the standard AI/ML solutions. This represents a missed opportunity for understanding patients' experience in an increasingly “connected” world. Thereby, SA and its ability to systematically review unstructured data is ready to overrun old limitations and produce higher-quality results.

In addition to these major benefits, several other perquisites have been highlighted in the literature. Firstly, data mining and SA may be used to explore the issues surrounding controversial research subjects, guidelines' changes or new recommendations in CV medicine (31).

Secondly, according to the Information Strategy for the National Health Service (NHS) in England, SA may be valuable for patients in facilitating choice of hospitals (32) by predicting, from free-text, “*a reasonably accurate assessment of patients*' *opinion about different performance aspects of a hospital*” (27).

Thirdly, online public testimonies carry classical indicators (such as self-reported quality of life indicators during and post treatment) and other relevant indicators (such as attitudes toward political legislation, loss of healthcare coverage, raising support, spreading awareness) that are difficult to capture by conventional means of self-reporting. Social listening can provide valuable feedback from patients and can help healthcare professionals and regulators to personalize and improve treatment regimens and improve public health surveillance strategies (33).

## A Synthesis of the Current Literature on SA in Cardiovascular Diseases

### Emotional Risk Factors for CVD

Poor emotion regulation was associated with CV risk in several studies (34, 35). Moreover, educational programs aimed at improving emotion regulation strategies among patients in cardiac rehabilitation proved to be feasible (36).

Whereas emotion regulation is a teachable skill that may play a role in preventing CVD, emotions must first be systematically recognized and documented before deciding whether the intervention of educational programs is appropriate. Several models of emotional recognition, capable of ensuring a systematic evaluation of sentiments, have been proposed based on eye-tracking (37–39), heart sound signals (40), cardiovascular response in daily life using the k-Nearest-Neighbor classifier (41), heart rate data collected from wearable devices (42), or even computational evaluation of facial expressions (43).

The Twitter platform was used on a large scale to assess the psychological language as a risk factor for atherosclerotic CAD by applying SA analysis (9). Hostility and chronic stress are known risk factors for CVD (44). All language patterns highlighting negative psychological traits (anger, negative-relationship, negative-emotion, and disengagement) emerged as significant risk factors [partial rs = 0.06, 95% confidence interval, or CI = (0.00, 0.11), to 0.12, 95% CI = (0.07,0.17)] for CAD mortality while the use of engagement words [*r* = −0.09, 95% CI = (−0.14, −0.04)] and positive-emotion words [partial *r* = –.05, 95% CI = (–.00, –.11)] appeared significantly protective. Surprisingly, a regression model “*based only on Twitter language*” predicts CAD mortality significantly better than a model with 10 common demographic, socioeconomic, and health risk factors (e.g., smoking, diabetes, hypertension, and obesity) (9).

A SA/ML methodology has been proposed to identify the relevant myocardial infarction (MI) emotional risk factors using Twitter data (10). Even if it seems unbelievable, the authors explore the possibility of screening tweets for MI risk factors as a tool to be used in preventive medicine. However, no results are yet provided.

Acute MI was repeatedly studied as an important consequence of stressful social disasters and social stress [e.g., the influence on MI of the death of a beloved (45), earthquakes (46), or war (47)]. In this context, a Korean team used the SA/ML algorithm of Semantria Lexalytics and managed to prove the effects of the Sewol Ferry Disaster on social stress by examining data from the top social media platforms used worldwide (YouTube, Twitter, and Facebook) (45).

Another way to harness the capabilities of SA was to analyze tweets in an attempt to find the relation between the sentiment of tweets and CVD in the counties along Interstate 20 in Texas (11). The sentiment of tweets from each region was determined by five classifiers (Naïve Bayes, Multinomial Naïve Bayes, Bernoulli Naïve Bayes, Support Vector, and Linear Support Vector) and was compared with the regional CVD rates. The Positive-to-Population rate is related to the CVD data map and Negative-to-Population rates have inverse relations to the CVD data map. This descriptive study highlights the potential of SA in epidemiological research, generating causal hypotheses and finding trends of diseases.

### Detecting Positive/Negative Attitudes of CV Patients Toward Their Disease in Order to Identify Strategies for Bettering Attitudes and Behaviors

Patients' attitude toward their disease may be an important drive for treatment adherence and a significant buffer of the impact of illness (48). However, the prevalence of positive attitudes toward the disease may often be low (49). SA is capable of contributing with systematized analysis and information on subjective attitudes (where traditional healthcare management is not able), fine-tuning deeper preventive strategies.

A method using SA was built to understand public health impact of CVD and patients' attitudes toward the disease in order to develop personalized therapeutic strategies depending on patients' adherence (12). Less than half of the tweets on CVD (45%) were found positive raising awareness on the importance of positive behavior change.

Addressing Behavioral change interventions could drastically reduce overall mortality from CVD (13). Behavior Change Techniques Taxonomy version 1 (BCTTv1) was applied to a sample of fitness and nutrition mobile apps and SA was used to identify which apps that support behavior change elicits a positive response from the users (13). StepsApp Pedometer had the highest percentage of positive tags while VeryFitPro had the lowest.

Various types of SA tools were used to examine the impact and improvement in diseases such as CVD, as SA contributes in designing strategies to improve patients understanding and behavior (50).

### Detection of Cardiac Arrhythmia

Computers can be trained to learn as humans do. Behadada et al. (14) proved that computers, as students, can learn from experts, textual data (scientific articles) and experience (experiments data). The authors introduced a novel method to define semi-automatically fuzzy partition rules to provide a powerful and accurate insight into the detection of cardiac arrhythmia. Fuzzy logic allows merging three completely different sources of knowledge by learning to define and integrate rule bases. The expert is invited to express his/her knowledge through linguistic (expert) rules. Moreover, the rules induced from data are called induced rules. Finally, the automated extraction of fuzzy partition rules from Pubmed articles identifies relevant arrhythmia insights and intuitions (mood described by text fragments) through grammar-based text extraction and SA. All extracted rules are merged into a unique knowledge base resulting in the definition of a common universe for the different knowledge domains. The evaluation carried out showed an accuracy rate of 93% and a high level of interpretability of 0.646 for the detection of cardiac arrhythmia.

Compared to the traditional ML solutions, besides an excellent accuracy, the approach proposed by Behadada et al. (14) comes with the major advantage of a high interpretability, as the computer is able to highlight all knowledge rules that led to a certain result.

### Triage of CV Patients

In a cardiovascular secondary prevention setting, the feasibility of using an ML program to triage and classify incoming SMS text messaging replies as requiring health professional review or not, was assessed and reported (15). The SMS messaging programs are a cost-efficient way for patients monitored in secondary prevention centers to regularly report their health status. However, the additional staff required to monitor and moderate the patients' SMS text messaging replies may negatively impact the cost-effectiveness of the SMS-based system. In order to reduce these costs, Lowres et al. (15) proposed five ML models (Naïve Bayes, OneVsRest, Random Forest Decision Trees, Gradient Boosted Trees, and Multilayer Perceptron) and an ensemble model for the automatic triaging of SMS replies. The Multilayer Perceptron model achieved the highest accuracy (AUC 0.86; 4.85% false negatives; and 4.63% false positives). After future validations against larger datasets, the authors are optimistic that the ML solution will significantly reduce staff workload.

### Feedbacks From Patients and Newspapers: Reviews on Drugs, Therapeutic Procedures, or Medical Devices

Medication and medical devices reviews are important to improve their quality, safety, adherence and use (51). Side effects may influence patients' adherence, thus pharmacovigilance is a key strategy to improve adherence (51).

The “SentiScrap” system applies SA through a hybrid approach (supervised machine learning and use of semantics through a tagged corpus) to identify opinions, comments, and polarity of the drugs prescribed for chronic-degenerative diseases (including hypertension medication), available in blogs and specialized websites in the Spanish language (16). Such a solution is of great help to health specialists as it reduces the time and effort to systematically search for patients' opinions, comments and experiences regarding the use of drugs, facilitating clinical decision making.

Medical devices' reviews were also considered for sentiment assessment. A lexicon-based SA was performed to pool together patients' experiences (fears, opinions, thoughts) from MyLVAD.com regarding their implanted left ventricular assist device (LVAD) (17). The results of the analysis indicate dominant positive sentiment {a net sentiment ratio [(number of positive words—number of negative words)/(number of total words)] of 2.1%} and a common use of the word “infection” (208 mentions) compared to other words denoting complications such as “stroke” (29 mentions), “bleeding” (30 mentions), and “thrombosis” or “clot” (32 mentions). This type of analysis might help to elucidate hidden, subjective segments of patients' health which factor into the objective measures of health.

Biotricity Inc. is a medical diagnostic and consumer healthcare tech company that is a leading producer of remote medical monitoring devices. Biotricity's main targets are chronic CVDs. With the help of the analytics firm InfoTrie Financial Solutions' Sentiment Analysis it was proved that Biotricity has been trending positively in the media, achieving a news buzz score of 10 out of 10, with a market sentiment score of 4.0 (18).

### SA Modules Integrated in New Technological Concepts for Monitoring CV Patients

A smart conceptual framework for monitoring patients with CV or diabetes was proposed (19). The concept respresents a hybrid healthcare system designed to merge distinct emerging computing techniques such as data mining, ML, Internet of Things (IoT), bio-sensors, SA, chatbots, contextual entity search, and granular computing. Bio-sensors and IoT are used for the continuous monitoring of the patient's health parameters and emergency notifications. SA is intended to mine social media and other online resources in order to keep the patient and the healthcare professional up to date regarding CV and diabetes updated informations. Data mining and ML are used for patient classification, diagnosis, and health predictions. This hybrid AI and smart framework may provide an effective and economical solution to CV and diabetes patients by minimizing various implicit and explicit medical expenses, optimizing the use of vital medical resources and manpower, and further enhancing the patient care.

## Challenges and Obstacles of Sentiment Analysis in Cardiovascular Health

SA is indeed a promising field that can add valuable insights to the traditional and objective measures of health and contribute to clinical decision making. However, SA is the hardest task in NLP as analyzing sentiments in an accurate manner is a difficult task even for humans.

Context and meaning play a crucial role in interpreting emotions. For instance, this Twitter post: “*Safe to say she may have been shocked to hear that the research does not suggest that high colesterol is a risk factor for heart disease*” was automatically classed as negative by an automated SA algorithm, due to the potentially negative concepts such as “shocked”, “high cholesterol”, “risk factor” and “heart disease” (52). However, the actual meaning is positive as the author is referring to the positive fact that the cited research does not incriminate negative associations. A major and mandatory challenge to SA techniques is to be able to integrate context (such as cultural, medical, political, legal, economic) and meaning. Moreover, in some cases, it is necessary to know much more than emotional polarity. For real life impact, SA algorithms should be equipped with the ability to categorize and organize subjective information, detect irony and sarcasm, comparisons, and emojis.

In general, the measure of how well humans annotators can decide on the same labels (inter-annotator agreement) is low when it comes to SA (53). Since machines learns from the data they are fed, SA models might not be as accurate as other types of classifiers. This challenge may be overcome after developing more rigorous definitions of emotional polarity and neutrality.

Another aspect to consider is that only 10% of individuals between the ages of 50–64 use social media sites such as Twitter (54). This limitation is worthy of consideration until social media platforms will become more popular among older patients.

One further obstacle is represented by the ethical implications of utilizing online publically available data from social media platforms for research purposes (55). Current regulations do not yet fully consider this aspect, although this is probably just a matter of time until ethical implications will be rigorously addressed and clarified.

## Conclusions

This paper introduced the readers to basic concepts surrounding medical SA and justified how SA can contribute to increasing quality in cardiovascular healthcare, emphasizing the need to invest more research into this new, promising and challenging domain. Our synthesis of the current literature on SA in CVDs proves its clinical potential. It also shows that the domain is only at the beginning. Many other clinical utilities, such as the assessment of emotional consequences of illness, patient-physician relationship, physician intuitions in CVD are not yet explored. These remain important research directions for the future, along with proposing detailed regulations for ethical implications, popularizing health social media and online expression among elders, developing more insightful definitions of emotional polarity and neutrality, and investing research into the discovery of powerful SA algorithms that are able to integrate global context and meaning.

## Author Contributions

ABu and ABr: conceptualization. ABu and IP: methodology and writing—review and editing. ABr, ABu, IP, MA, and OG: resources and writing—original draft preparation. ABr, OG, and MA: supervision. All authors have read and agreed to the published version of the manuscript.

## Conflict of Interest

ABr and IP were employed by GreenSoft Ltd. The remaining authors declare that the research was conducted in the absence of any commercial or financial relationships that could be construed as a potential conflict of interest.

## Publisher's Note

All claims expressed in this article are solely those of the authors and do not necessarily represent those of their affiliated organizations, or those of the publisher, the editors and the reviewers. Any product that may be evaluated in this article, or claim that may be made by its manufacturer, is not guaranteed or endorsed by the publisher.

## References

[1] Ligthart A Catal C Tekinerdogan B. Systematic reviews in sentiment analysis: a tertiary study. Artif Intell Rev. (2021) 54:4997–5053. 10.1007/s10462-021-09973-3

[2] Briganti G Le Moine O. Artificial intelligence in medicine: today and tomorrow. Front Med. (2020) 7:27. 10.3389/fmed.2020.00027 32118012 PMC7012990

[3] Ghassemi MM Al-Hanai T Raffa JD Mark RG Nemati S Chokshi FH. How is the doctor feeling? ICU provider sentiment is associated with diagnostic imaging utilization. Annu Int Conf IEEE Eng Med Biol Soc. (2018) 2018:4058–64. 10.1109/EMBC.2018.8513325 30441248

[4] Gohil S Vuik S Darzi A. Sentiment analysis of health care tweets: review of the methods used. JMIR Public Health Surveill. (2018) 4:e43–43. 10.2196/publichealth.5789 29685871 PMC5938573

[5] WHO. Cardiovascular diseases (CVDs). WHO (2021). Available online at: https://www.who.int/news-room/fact-sheets/detail/cardiovascular-diseases-(cvds) (accessed January 15, 2022).

[6] Eberly LA Khatana SAM Nathan AS Snider C Julien HM Deleener ME Adusumalli S Telemedicine outpatient cardiovascular care during the COVID-19 pandemic. (2020) 142:510–2. 10.1161/CIRCULATIONAHA.120.048185 32510987 PMC9126131

[7] Korkontzelos I Nikfarjam A Shardlow M Sarker A Ananiadou S Gonzalez GH. Analysis of the effect of sentiment analysis on extracting adverse drug reactions from tweets and forum posts. J Biomed Inform. (2016) 62:148–58. 10.1016/j.jbi.2016.06.007 27363901 PMC4981644

[8] Nikfarjam A Sarker A O'Connor K Ginn R Gonzalez G. Pharmacovigilance from social media: mining adverse drug reaction mentions using sequence labeling with word embedding cluster features. J Am Med Inform Assoc. (2015) 22:671–81. 10.1093/jamia/ocu041 25755127 PMC4457113

[9] Eichstaedt JC Schwartz HA Kern ML Park G Labarthe DR Merchant RM. et al. Psychological language on twitter predicts county-level heart disease mortality. Front Psychol. (2015) 26:159–69. 10.1177/0956797614557867 25605707 PMC4433545

[10] Hemalatha R Monicka M. Sentiment analysis on myocardial infarction using tweets data. Int J Comput Sci Technol. (2018) 9:61–5. Available online at: http://www.ijcst.com/vol9/issue4/12-m-b-monicka.pdf

[11] Medina Sada D Mengel S Gittner LS Khan H Rodriguez MAP Vadapalli R. A preliminary investigation with twitter to augment cvd exposome research. In: Proceedings of the Fourth IEEE/ACM International Conference on Big Data Computing, Applications and Technologies. New York, NY: Association for Computing Machinery (2017). p. 169–78. Available online at: https://dl.acm.org/doi/pdf/10.1145/3148055.3148074

[12] Verma L Sapra V. Semantic analysis of cardiovascular disease sentiment in online social media. In: Proceedings of International Conference on Advancements in Computing & Management (ICACM) (2019). Available online at: https://papers.ssrn.com/sol3/papers.cfm?abstract_id=3462426

[13] Pimenta F Lopes L Gonçalves F Campos P. Designing positive behavior change experiences: a systematic review and sentiment analysis based on online user reviews of fitness and nutrition mobile applications. In: 19th International Conference on Mobile and Ubiquitous Multimedia. New York, NY: Association for Computing Machinery (2020). p. 152–61. Available online at: https://dl.acm.org/doi/10.1145/3428361.3428403

[14] Behadada O Trovati M Chikh MA Bessis N. Big data-based extraction of fuzzy partition rules for heart arrhythmia detection: a semi-automated approach. Concurrency Comput Pract Exp. (2016) 28:360–73. 10.1002/cpe.3428

[15] Lowres N Duckworth A Redfern J Thiagalingam A Chow CK. Use of a machine learning program to correctly triage incoming text messaging replies from a cardiovascular text–based secondary prevention program: feasibility study. JMIR Mhealth Uhealth. (2020) 8:e19200. 10.2196/19200 32543439 PMC7327598

[16] Pérez KC Sánchez-Cervantes JL del Pilar Salas-Zárate M LÁR Hernández L Rodríguez-Mazahua A. Sentiment analysis approach for drug reviews in Spanish Res Comput Sci. (2020) 149:43–51.

[17] Austin MA Saxena A O'Malley TJ Maynes EJ Moncure H Ott N. Computational sentiment analysis of an online left ventricular assist device support forum: positivity predominates. Ann Cardiothorac Surg. (2020) 10:375–82. 10.21037/acs-2020-cfmcs-fs-11 34159118 PMC8185377

[18] Markets E. Emerging Markets Report: What's the Big Buzz on Biotricity? (2020). Available online at: https://www.globenewswire.com/news-release/2020/02/10/1982225/0/en/Emerging-Markets-Report-What-s-the-Big-Buzz-on-Biotricity.html (accessed January 15, 2022).

[19] Sharma M Singh G Singh R. An advanced conceptual diagnostic healthcare framework for diabetes and cardiovascular disorders. arXiv preprint arXiv:1901.10530 (2019). 10.4108/eai.19-6-2018.154828

[20] Denecke K Deng Y. Sentiment analysis in medical settings: new opportunities and challenges. Artif Intell Med. (2015) 64:17–27. 10.1016/j.artmed.2015.03.006 25982909

[21] Kaity M Balakrishnan V. Sentiment lexicons and non-english languages: a survey. Knowl Inf Syst. (2020) 62:4445–80. 10.1007/s10115-020-01497-6

[22] Lo SL Cambria E Chiong R Cornforth D. Multilingual sentiment analysis: from formal to informal and scarce resource languages. Artif Intell Rev. (2017) 48:499–527. 10.1007/s10462-016-9508-4

[23] Aqlan AAQ Manjula B Lakshman Naik R. A Study of Sentiment Analysis: Concepts, Techniques, and Challenges. Singapore: Springer Singapore (2019). p. 147–62.

[24] Boiy E Moens M-F. A machine learning approach to sentiment analysis in multilingual web texts. Inf Retr Boston. (2009) 12:526–58. 10.1007/s10791-008-9070-z

[25] Tennant C McLean L. The impact of emotions on coronary heart disease risk. J Cardiovasc Risk. (2001) 8:175–83. 10.1177/174182670100800309 11455850

[26] Settanni M Marengo D. Sharing feelings online: studying emotional well-being via automated text analysis of facebook posts. Front Psychol. (2015) 6:1045. 10.3389/fpsyg.2015.01045 26257692 PMC4512028

[27] Greaves F Ramirez-Cano D Millett C Darzi A Donaldson L. Use of sentiment analysis for capturing patient experience from free-text comments posted online. J Med Internet Res. (2013) 15:e239. 10.2196/jmir.2721 24184993 PMC3841376

[28] Sinnenberg L DiSilvestro CL Mancheno C Dailey K Tufts C Buttenheim AM. Twitter as a potential data source for cardiovascular disease research. JAMA Cardiol. (2016) 1:1032–6. 10.1001/jamacardio.2016.3029 27680322 PMC5177459

[29] Medhat W Hassan A Korashy H. Sentiment analysis algorithms and applications: a survey. Ain Shams Eng J. (2014) 5:1093–113. 10.1016/j.asej.2014.04.011

[30] Wang G Xie M Ma J Guan J Song Y Wen Y. Is co-infection with influenza virus a protective factor of COVID-19? SSRN Electronic J. (2020). 10.2139/ssrn.3576904

[31] Gouda P Das D Clark A Ezekowitz JA. The impact and implications of twitter for cardiovascular medicine. J Card Fail. (2017) 23:266–7. 10.1016/j.cardfail.2016.12.005 28010999

[32] Health DO. The Power of Information: Putting All of Us in Control of the Health and Care Information We Need. London: Department of Health (2012).

[33] Clark EM. Applications in Sentiment Analysis Machine Learning for Identifying Public Health Variables Across Social Media. The University of Vermont State Agricultural College, Burlington, United States (2019). Available online at: https://scholarworks.uvm.edu/graddis/1006/

[34] Roy B Riley C Sinha R. Emotion regulation moderates the association between chronic stress and cardiovascular disease risk in humans: a cross-sectional study. Stress. (2018) 21:548–55. 10.1080/10253890.2018.1490724 30084712 PMC6367063

[35] Besharat MA Ramesh S. The relationship between worry and anger rumination with adjustment problems to heart disease: The mediating role of difficulties in emotion regulation. Heart and Mind. (2017) 1:141. 10.4103/hm.hm_7_18

[36] Wierenga KL Fresco DM Alder M Moore SM. Feasibility of an emotion regulation intervention for patients in cardiac rehabilitation. West J Nurs Res. (2021) 43:338–46. 10.1177/0193945920949959 32814517 PMC9116464

[37] Lim JZ Mountstephens J Teo J. Emotion recognition using eye-tracking: taxonomy, review and current challenges. Sensors. (2020) 20:2384. 10.3390/s20082384 32331327 PMC7219342

[38] Tarnowski P Kołodziej M Majkowski A Rak RJ. Eye-Tracking Analysis for Emotion Recognition. Comput Intell Neurosci. (2020) 2020:2909267. 10.1155/2020/2909267 32963512 PMC7492682

[39] Schurgin M Nelson J Iida S Ohira H Chiao J Franconeri S. Eye movements during emotion recognition in faces. J Vis. (2014) 14:14–14. 10.1167/14.13.14 25406159

[40] Xiefeng C Wang Y Dai S Zhao P Liu Q. Heart sound signals can be used for emotion recognition. Sci Rep. (2019) 9:6486. 10.1038/s41598-019-42826-2 31019217 PMC6482302

[41] Jo Y Lee H Cho A Whang M. Emotion Recognition Through Cardiovascular Response in Daily Life Using KNN Classifier. Singapore: Springer Singapore (2018). p. 1451–6.

[42] Shu L Yu Y Chen W Hua H Li Q Jin J. Wearable emotion recognition using heart rate data from a smart bracelet. Sensors. (2020) 20:718. 10.3390/s20030718 32012920 PMC7038485

[43] Tarnowski P Kołodziej M Majkowski A Rak RJ. Emotion recognition using facial expressions. Procedia Comput Sci. (2017) 108:1175–84. 10.1016/j.procs.2017.05.025

[44] Menezes AR Lavie CJ Milani RV O'Keefe J Lavie TJ. Psychological risk factors and cardiovascular disease: is it all in your head? Postgrad Med. (2011) 123:165–76. 10.3810/pgm.2011.09.2472 21904099

[45] Mostofsky E Maclure M Sherwood JB Tofler GH Muller JE Mittleman MA. Risk of acute myocardial infarction after the death of a significant person in one's life: the determinants of myocardial infarction onset study. Circulation. (2012) 125:491–6. 10.1161/CIRCULATIONAHA.111.061770 22230481 PMC3397171

[46] Suzuki S Sakamoto S Koide M Fujita H Sakuramoto H Kuroda T. Hanshin-Awaji earthquake as a trigger for acute myocardial infarction. Am Heart J. (1997) 134:974–7. 10.1016/S0002-8703(97)80023-3 9398112

[47] Kim YH Her AY Rha SW Choi BG Shim M Choi SY. Routine angiographic follow-up versus clinical follow-up after percutaneous coronary intervention in acute myocardial infarction yonsei. Med J. (2017) 58:720–30. 10.3349/ymj.2017.58.4.720 28540983 PMC5447101

[48] Dushad R Mintu M Samaksha PB Basavana GH. A study of drug attitude and medication adherence and its relationship with the impact of illness among the mentally ill. Arc Clin Psychiatr. (2019) 46:85–8. 10.1590/0101-60830000000201

[49] Assunção SC Fonseca AP Silveira MF Caldeira AP Pinho LD. Knowledge and attitude of patients with diabetes mellitus in primary health care. Escola Anna Nery. (2017) 21. 10.1590/2177-9465-ean-2017-0208

[50] Bhoi D Thakkar A. 12. Impact of sentiment analysis tools to improve patients' life in critical diseases. In: Srivastava R Mallick PK Rautaray SS Pandey M, editors. Computational Intelligence for Machine Learning and Healthcare Informatics. Berlin; Boston, MA: De Gruyter (2020). p. 239–52. Available online at: https://www.degruyter.com/document/doi/10.1515/9783110648195-012/html

[51] Blenkinsopp A Bond C Raynor DK. Medication reviews. Br J Clin Pharmacol. (2012) 74:573–80. 10.1111/j.1365-2125.2012.04331.x 22607195 PMC3477324

[52] Kennedy K. Does Automated Sentiment Analysis Work for Studying Healthcare Conversation? Creation Knowledge (2015). Available online at: https://creation.co/knowledge/automated-sentiment-analysis-healthcare-conversation/

[53] Bobicev V Sokolova M. Inter-annotator agreement in sentiment analysis: machine learning perspective. In: Proceedings of the International Conference Recent Advances in Natural Language Processing, RANLP 2017. Varna: Incoma Ltd (2017). p. 97–102. Available online at: https://www.acl-bg.org/proceedings/2017/RANLP%202017/pdf/RANLP015.pdf

[54] Duggan M Brenner J. The Demographics of Social Media Users, 2012. Washington, DC: Pew Research Center's Internet & American Life Project (2013).

[55] Gouda P Das D Clark A Ezekowitz JA. The impact and implications of twitter for cardiovascular medicine. J Card Fail. (2016) 23:266–7. Available online at: https://www.onlinejcf.com/article/S1071-9164(16)31242-8/fulltext 28010999 10.1016/j.cardfail.2016.12.005

